# Clinical presentations and outcomes of patients with Ebola virus disease in Freetown, Sierra Leone

**DOI:** 10.1186/s40249-016-0195-9

**Published:** 2016-11-03

**Authors:** Ying-Jie Ji, Xue-Zhang Duan, Xu-Dong Gao, Lei Li, Chen Li, Dong Ji, Wen-Gang Li, Li-Fu Wang, Yu-Hua Meng, Xiao Yang, Bin-Fang Ling, Xue-Ai Song, Mei-Lei Gu, Tao Jiang, She-Ku M. Koroma, James Bangalie, Hui-Juan Duan

**Affiliations:** 1 Chinese Military Medical Aid Team in Sierra Leone, The Beijing 302 Hospital, 100 XI-SIHUAN Middle Road, Beijing, 100039 China; 2 China CDC Mobile Laboratory, Chinese Academy of Science, Beijing, 100039 China; 3 Jui Government Hospital, Freetown, Sierra Leone

**Keywords:** Ebola virus disease, Ebola virus, Mortality

## Abstract

**Background:**

Clinical and laboratory data were collected and analysed from patients with Ebola virus disease (EVD) in Jui Government Hospital in Freetown, Sierra Leone, where patients with EVD were received and/or treated from October 1, 2014 to March 21, 2015 during the West Africa EVD outbreak.

**Methods:**

The study admitted 285 patients with confirmed EVD and followed them up till the endpoint (recovery or death). EVD was confirmed by quantitative RT-PCR assays detecting blood Ebola virus (EBOV).

**Results:**

Among the 285 lab-confirmed EVD cases in Jui Government Hospital, 146 recovered and 139 died, with an overall survival rate of 51.23 %. Patients under the age of 6 years had a lower survival rate (37.50 %). Most non-survivors (79.86 %) died within 7 days after admission and the mean hospitalization time for non-survivors was 5.56 ± 6.11 days. More than half survivors (63.69 %) turned blood EBOV negative within 3 weeks after admission and the mean hospitalization time for survivors was 20.38 ± 7.58 days. High blood viral load (≥10^6^ copies/ml) was found to be predictive of the non-survival outcome as indicated by the Receiver Operating Characteristic (ROC) curve analysis. The probability of patients’ survival was less than 15 % when blood viral load was greater than 10^6^ copies/ml. Multivariate analyses showed that blood viral load (*P* = 0.005), confusion (*P* = 0.010), abdominal pain (*P* = 0.003), conjunctivitis (*P* = 0.035), and vomiting (*P* = 0.004) were factors independently associated with the outcomes of EVD patients.

**Conclusions:**

Most death occurred within 1 week after admission, and patients at the age of 6 or younger had a lower survival rate. Most surviving patients turned blood EBOV negative within 1–4 weeks after admission. Factors such as high blood viral load, confusion, abdominal pain, vomiting and conjunctivitis were associated with poor prognosis for EVD patients.

**Electronic supplementary material:**

The online version of this article (doi:10.1186/s40249-016-0195-9) contains supplementary material, which is available to authorized users.

## Multilingual abstracts

Please see Additional file 1 for translations of the abstract into the six official working languages of the United Nations.

## Background

Ebola virus disease (EVD), previously known as Ebola haemorrhagic fever, is a rare and deadly disease caused by infection with one of the Ebola virus strains. A large-scale outbreak of haemorrhagic fever occurred in southern Sudan between June and November 1976. It was transmitted by close personal contact and by use of contaminated needles and syringes in hospitals/clinics [1]. This outbreak led to the further recognition of the disease, which was subsequently named Ebola haemorrhagic fever. Since then, outbreaks have occurred sporadically in Africa.

A 2014 EVD outbreak was the largest in scale in history, affecting multiple countries in West Africa. In the 2014 outbreak, the first lab-confirmed EVD patient was reported in May, 2014 in Guinea and since then the Zaire Ebola Virus (ZEBOV) has rapidly spread across Sierra Leone and to other West Africa countries. From March 2014 to December 27, 2015,there were 28 601 reported EVD infections (including confirmed, probable, and suspected) and 11 300 reported deaths in West Africa. A total of 853 confirmed infections and 494 deaths of health care workers were reported in Guinea, Liberia, and Sierra Leone [2].

As of December 23, 2015, the Sierra Leone National Ebola Response Center (NERC) reported a cumulative total of 14 339 confirmed EVD cases with 3 955 deaths (excluding probable and suspected cases) [3]. Situated on the Atlantic coast, Freetown is the capital and the largest city of Sierra Leone, and is densely populated with over 1 million people. Due to the heavy population, Freetown and its surrounding western region was the most affected area in this epidemic. As of December 23, 2015, 5 500 confirmed cases had been reported in this region, accounting for 38.36 % of the country’s total EVD reported cases [3].

EVD imposed a significant economic burden on the West African countries affected. Some studies suggest that due to EVD deaths, life expectancy may have declined in Liberia and Sierra Leone to a new low since 2001–2003 [4]. This dramatic healthcare crisis, coupled with human rights and global security concerns, underscored the urgent need for developing resilient healthcare systems, and called for the domestic and international aides and investments in these African countries [5].

The clinicians of the Chinese Medical Team (CMT) managed EVD patients in an Ebola Holding and Treatment Center in Jui Government Hospital, which is also known as Sierra Leone-China Friendship Hospital. Being one of the best hospitals in Freetown, Jui Government Hospital received 773 suspected EVD patients during the period of October 1, 2014 and March 21, 2015, of whom 285 were confirmed infected with the virus. All of the CMT clinicians were from Beijing 302 Hospital, the largest specialized hospital in China for infectious disease treatment. The same hospital had successfully managed and controlled the outbreak of Severe Acute Respiratory Syndrome (SARS), A/H1N1 influenza, and some other public health emergencies in China.

In this study, we described the clinical presentations, clinical courses, and the treatment outcomes of all EVD patients admitted in Jui Government Hospital for care. We hope this paper provide further understanding and insights into pathophysiology, clinical manifestations and treatment impact of end outcomes of EVD.

## Methods

### Patients and data collection

A retrospective, observational study was conducted using data collected from all patients with confirmed EVD who were admitted to the Holding and Treatment Center of Jui Government Hospital from October 1, 2014 to March 21, 2015. Diagnosis of EVD was made in accordance with the criteria set by the World Health Organization (WHO) in the Standard Operating Procedures (SOP) for Managing EVD.

Because Jui Government Hospital was designated as an Ebola Holding Center (EHC) on October 1, 2014, but was not approved as an Ebola Treatment Center (ETC) until January 1, 2015, 152 confirmed EVD patients received at this hospital between October 1, 2014 and January 1, 2015 were immediately transferred to other treatment centres once confirmed. After January 1, 2015, all confirmed patients except pregnant women were treated in Jui Government Hospital; the confirmed EVD patients who were pregnant women were transferred to a designated ETC (P.T.S.1 Ebola Treatment Center). We carried out follow-up studies and data collections from all confirmed patients (both the patients treated in Jui Government Hospital and those transferred to other hospitals) before we finished our mission in March 2015. Data collected included observations such as the duration of hospitalization, the date when blood EBOV turned negative, and the endpoint (recovery or death). The determination of recovery was based on the clinical presentations and the interpretation of laboratory findings. Discharge from hospitalization was considered when the following criteria were met: 1) three or more days without fever or any other significant symptom, 2) significant improvement in clinical presentations, 3) a relatively good general condition, and 4) a negative PCR result for blood EBOV on the third day of being asymptomatic. If a patient continued to suffer symptoms or their condition was not improving, but it was suspected to be unrelated to EVD, then two blood EBOV tests were carried out 48 h apart, with at least one test being done 3 days or more after the onset of symptoms. If both test results were negative, patients were discharged or referred to a normal hospital for further care.

The study protocol adhered to the Declaration of Helsinki, and the ethical clearance was obtained from 302 Military Hospital Medical Ethics Committee and the Sierra Leone Ethics and Scientific Review Committee, respectively. The patients were routinely evaluated for clinical presentations. Data collected included vital signs at admission, medical history, time points when blood EBOV turned negative, durations of hospital stay, and outcome.

### EBOV diagnosis and viral load assays

Within 24 h upon admission, EBOV detection assays were carried out via quantitative RT-PCR using whole blood samples. If the patient died quickly and the blood samples were not collected due to time constraints or poor venous access after death, EBOV detection was carried out using oral swab samples from corpses. Samples were collected at 16:00 o’clock every day and results were generated within 6 h. Samples were obtained using Jui Government Hospital’s collection and processing protocols, which was described in the emergency-response guidelines established by the Sierra Leone Ministry of Health and Sanitation.

Diagnosis testing for EBOV was performed by China CDC Mobile Laboratory. Total RNA was extracted from patient peripheral blood samples or swab samples in Bio-safety Level 3 (BSL-3) facilities. Ebola viral RNA was then detected using Detection Kit for Ebola virus subtype Zaire RNA (Puruikang Biotech Co. Ltd, with PCR Fluorescence Probing) according to the manufacturer’s recommendations [6]. For quantification purposes, the amplicon concentrations were converted to copies of EBOV per milliliter (method provided by China CDC Mobile Laboratory).

### Treatment protocol

The care protocols for confirmed Ebola cases before and after June 1, 2014 were similar and were in accordance with the SOP of WHO and the Ministry of Health of Sierra Leone. The treatment protocol was as follows: All adult patients received 10 mg of vitamin K and 120 mg of sodium artesunate immediately upon admission. After 24 h all adult patients with confirmed EVD received 2 g of ceftriaxone every 24 h, 500 mg of metronidazole every 8 h, 500-1 000 ml of Ringer’s lactate every 12 h, and 500-1 000 ml of dextrose saline (5 % and 0.9 %, respectively) every 12 h. All the above medications were administered intravenously. Adult patients also received a 20 mg zinc sulfate tablet daily, a 400 mg acetaminophen tablet every 12 h, and 8 mg of ondansetron injection intravenously as needed for nausea or vomiting. After the first 3 days, continuing therapy included a 400 mg metronidazole tablet every 12 h for 7 days, a 500 mg cefuroxime tablet every 12 h for 5 days, and a 400 mg acetaminophen tablet every 12 h. The protocol for children was similar but the dosage was adjusted according to their body weight. Oral rehydration solution and juice were provided on an as needed basis. We transfused more fluid to the patients with profound GI losses and those unable to take oral fluids or medications. Currently, early supportive care, empiric antibiotic therapy, and maintenance of water and electrolyte balance are the basic interventions to treat EVD due to the lack of specific anti-EBOV drugs. Every ETC designed their treatments protocol according to the protocols for viral haemorrhagic fever under the urgent interim guidance for case management established by the WHO and endorsed by the Ministry of Health, which were similar.

### Statistical analyses

Statistical analyses were performed with the aid of SPSS, Version 17.0. (SPSS Inc., Chicago, IL, USA). The overall survival rate was compared using Log-Rank Test. Univariate and multivariate analyses were carried out using the logistic regression model. The inter-group comparisons were performed using Chi Square Test. Receiver Operating Characteristic (ROC) curve was plotted using the log_10_ value of blood EBOV viral load and the survival rate. All the statistical tests were two-tailed, and a *P*-value of less than 0.05 was considered statistically significant.

## Results

### EVD patient characteristics

A total of 773 suspected EVD patients were admitted to Jui government hospital, among which 285 were confirmed with EVD. All patients with confirmed EVD received supportive treatment and were followed up to the endpoint (recovery or death). Among the confirmed EVD patients, 152 were transferred to other treatment centres and 133 treated in our centre. Of the total confirmed, 139 were female and 146 were male, 146 recovered and 139 died, with an overall survival rate of 51.23 % (females 54.68 %, males 47.95 %). No significant difference was found between the survival rates of females and males (*P* = 0.2558).

The average age of the EVD patients was 29.20 ± 16.37 years, and the median age was 28 years, interquartile range were 19 years and 38 years (IQR, 19–38). The youngest patient was 1 month old, and the oldest 80 years old. The ages were separated by the following groups: 62 patients (21.75 %) were under the age of 16, 144 patients (50.53 %) between 16 and 35, 60 patients (21.05 %) between 36 and 60, and 19 patients (6.67 %) above the age of 60. The median age was 29 (IQR, 17–40) for the non-survivors and 27 (IQR, 17–35) for the survivors.

The mean hospitalization time for survivors was 20.38 ± 7.58 days, and the median was 19 (IQR, 15–24) days. The surviving patients were discharged after they had been asymptomatic for 48 h, and they had been tested negative for blood EBOV using RT-PCR assay. The mean hospitalization time for non-survivors was 5.56 ± 6.11 days, and the median was 4 (IQR, 3–6) days.

### Survivors timeline to negative blood EBOV diagnosis result

**Fig. 1:**
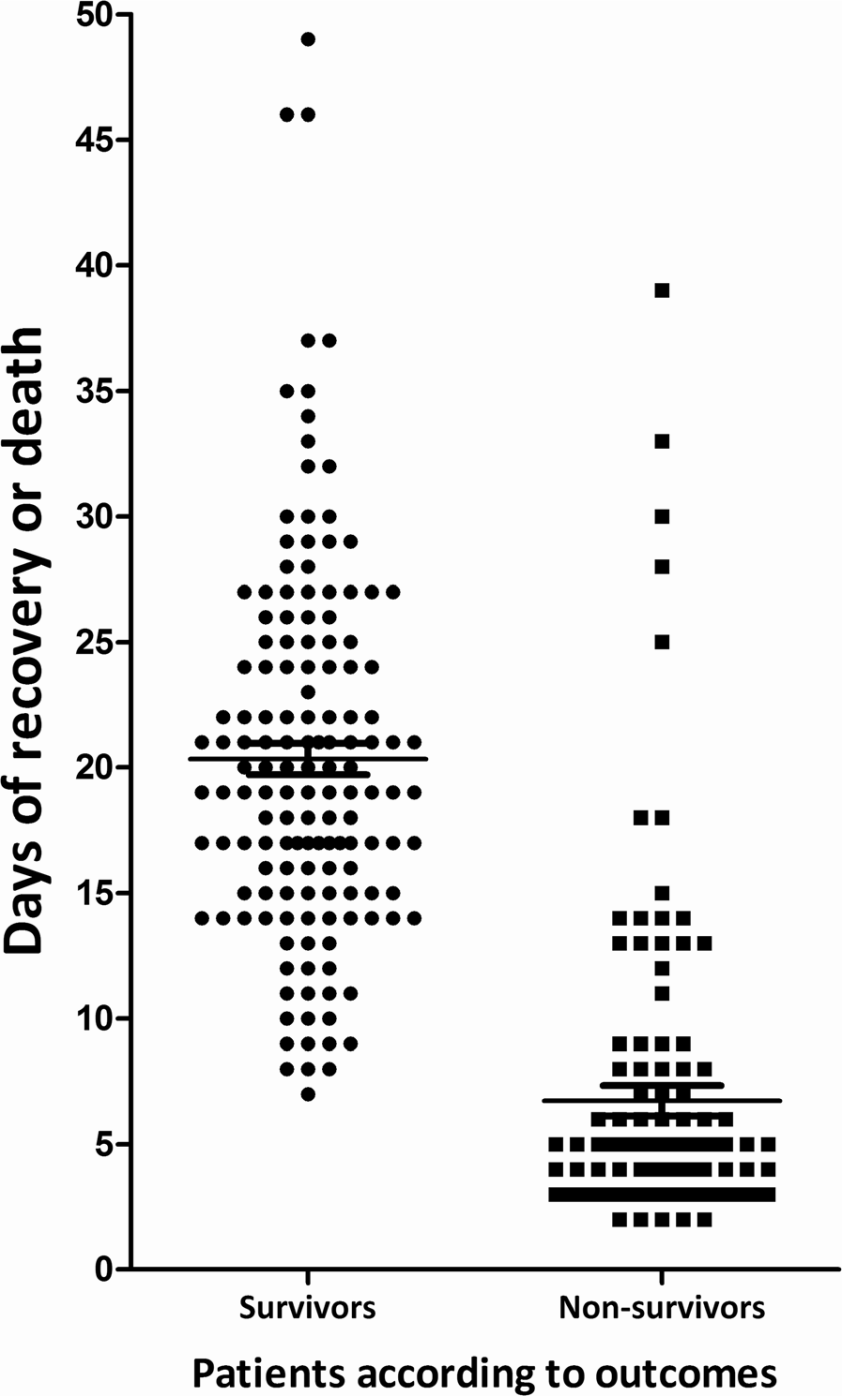
The time lapsed before survivors turned negative for blood EBOV, and the time lapsed before non-survivors died. All patients were confirmed with EVD in Sierra Leone

We investigated how long it took for the blood EBOV to turn negative in the surviving patients. All 146 survivors were followed up from the time of diagnosis (when blood EBOV was first detected) to the time of recovery. The median time for a surviving EVD patient to become blood EBOV negative was 20.31 ± 7.62 days, and the range was from 7 days to 49 days. Of the total number of survivors observed, 63.69 % (93 cases) turned negative for blood EBOV within 3 weeks after the diagnosis, and 87.67 % (128 cases) turned negative 4 weeks after the diagnosis (Fig. 1).

### Non-survivors hospitalization time

Among the 285 EVD patients who received treatment, 139 died (the fatality rate 48.77 %). Of the 139 non-surviving patients, 60.43 % died within 4 days after admission, 79.86 % died during the first week, and more than 90.00 % died within 2 weeks (Fig. 1).

### Age stratified survival rate

**Fig. 2:**
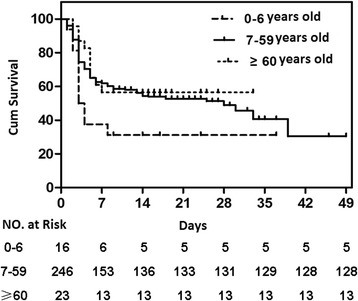
Comparison of the survival rates of EVD Patients in different age groups. (0–6 group compared to 7–59 group, *P* = 0.0424; 0–6 group compared to 60+ group, *P* = 0.0447; 7–59 group compared to 60+ group, *P* = 0.6621)

To investigate the survival rate of EVD patients in different age groups, the 285 EVD patients were divided into three groups (age 0–6, age 7–59, and ≥60 years). The results showed that the survival rate for group 0–6 was statistically lower than that of group 7–59 (*P* = 0.0424) or group ≥60 (*P* = 0.0447) (Fig. 2). But the survival rates for group 7–59 and group ≥60 were not statistically different (*P* = 0.6621, Kaplan–Meier Estimate) (Fig. 2). In addition, we categorized the patients using the median age (28 years) or 40 years as the cutoffs, and applied the same statistical analysis, but no statistically significant difference was found in the survival rates between groups.

### The viral load and the fatality rate of patients with EVD

**Fig. 3:**
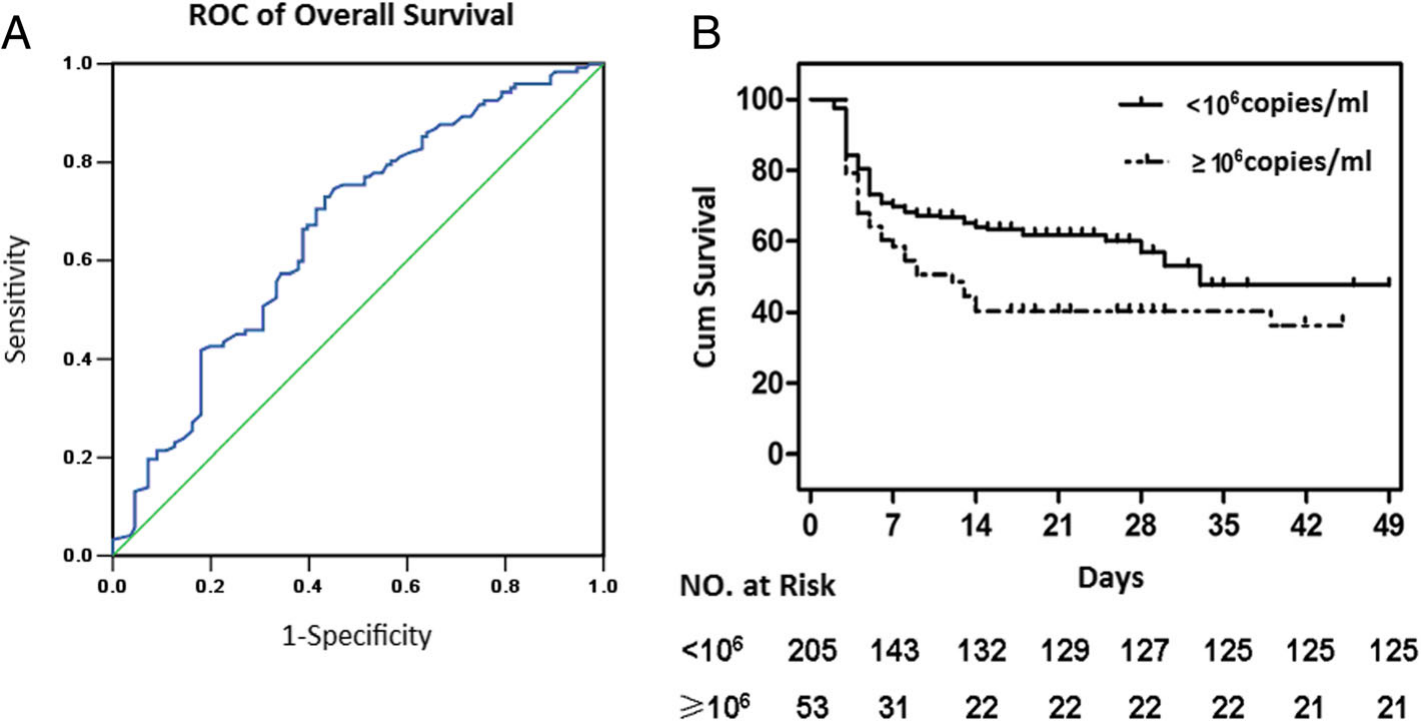
**a** Viral load as a predicator for the outcomes, Receive Operating Characteristic (ROC) curve. **b** Comparison of the survival rates of EVD Patients with different viral loads. (≥10^6^ group compared to <10^6^ group:*P* = 0.0066)

We also looked into whether high viral load in the blood was an indicator for high fatality rate. A Receiver Operating Characteristic (ROC) curve was plotted using the blood viral values and the survival outcomes of the EVD patients. Among the 285 patients, 27 died within 24 h of admission. Such patients were tested positive using only oral swab samples, and were excluded from this analysis. A total of 258 patients with detectable blood EBOV virus were included in the ROC curve analysis. The numbers of EBOV copies were converted to log_10_ values for further analysis. The results showed that the viral loads had a high predictive power of patients’ outcome (*P* < 0.001). The Areas Under Operator Curve (AUOC) were 0.663 (95 % *CI*: 0.593–0.733). From the ROC curve, when the log_10_ viral value was greater than 5, the probability of patients’ survival was less than 40 %; and when it was greater than 6, the probability of patients’ survival was less than 15 %. These results suggested that the viral loads can be used as a potential prognostic biomarker for EVD patients (Fig. 3a).

We selected 10^6^ copies/ml as the cutoff value to divide the patients into two groups (EBOV ≥ 10^6^copies/ml and < 10^6^ copies/ml), and applied statistical analysis (Fig. 3b). Kaplan–Meier Estimate showed that when the blood EBOV titer was greater than 10^6^ copies/ml, the fatality rate was 60.37 % -remarkably higher than that of the group with less than 10 ^6^ copies/ml viral load (39.02 %) (*P* = 0.0066).

### Comparison of survivors and non-survivors about clinical presentations

**Fig. 4:**
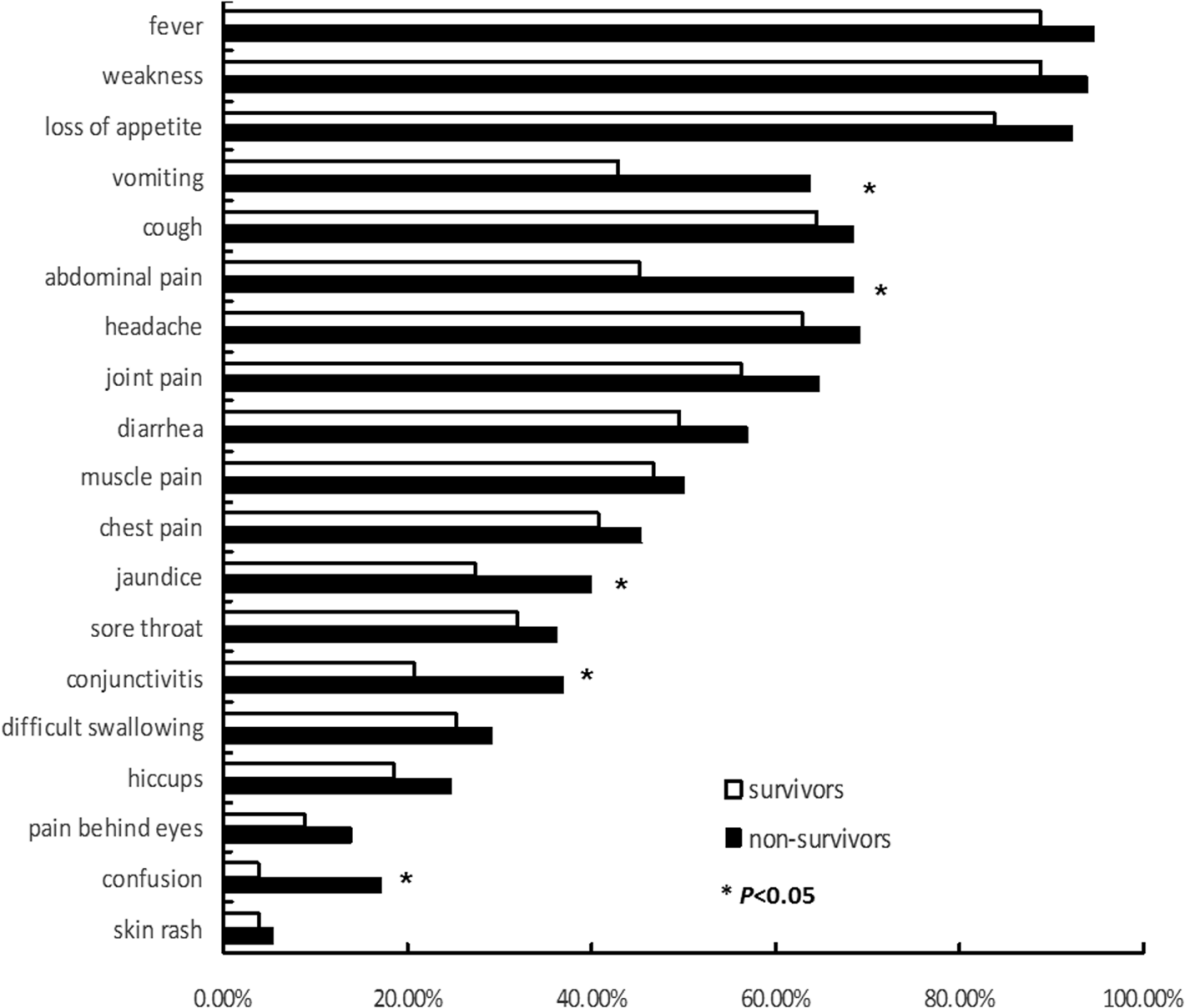
Comparison of symptoms between survivors and non-survivors

We analysed the clinical presentations of the EVD patients at the time of admission, of which 20 of the 285 patients were excluded due to incomplete data, making the total number of patients studied and included in the analysis to 265. Common findings of the clinical manifestations included fever (91.70 % of the patients), weakness (91.32 %), loss of appetite (87.92 %), cough (66.42 %), headache (66.04 %), joint pain (60.38 %), abdominal pain (56.60 %), vomiting (53.21 %), diarrhoea (53.21 %), muscle pain (48.30 %), chest pain (43.02 %), sore throat (33.96 %), jaundice (33.58 %), conjunctivitis (28.68 %), hiccups (21.51 %), bleeding (12.30 %), pain behind eyes (11.32 %), confusion (8.30 %), and skin rash (4.53 %). A number of variables between survivors and non survivors were shown to be significantly different amongst the EVD positive patients with known outcomes including vomiting (*P* < 0.001), abdominal pain (*P* = 0.020), jaundice (*P* < 0.001), conjunctivitis (*P* = 0.004), and confusion (*P* < 0.001) (Fig. 4).

### Multivariate analysis of factors associated with patients’ survival

To further explore which factors were associated with the survival of EVD patients, a number of factors, including a variety of symptoms (absent OR present), age (years), gender (male OR female), viral load (log_10_ copies/ml), and treatment outcomes (survival OR death) were included in the univariate and multivariate analyses (Table 1). The univariate analysis showed that vomiting, abdominal pain, jaundice, conjunctivitis, confusion, and the viral load (*P* < 0.15) were good candidates for the final Logistic Regression Model. The multivariate analyses showed that higher viral load, severe confusion, vomiting, abdominal pain, and conjunctivitis indicated poor prognosis in EVD patients (Table 1).

**Table 1:** The association of the outcomes with the symptoms and the viral load in EVD patients

Variable	Univariate *P*	Multivariate		Variable	Univariate *P*	Multivariate	
		*P*-value	*OR* (95 % *CI*)			*P*-value	OR (95.0 % *CI*)
Gender	0.368			Age	0.641		
Fever	0.223			Diarrhea	0.249		
Weakness	0.201			Cough	0.524		
Chest pain	0.469			Headache	0.300		
Difficult swallowing	0.478			Sore throat	0.481		
Pain behind eyes	0.210			Muscle pain	0.619		
Skin rash	0.520			Hiccups	0.237		
Loss of appetite	0.165			Joint pain	0.177		
Confusion	*0.000	*0.010	4.150 (1.401–12.293)	Jaundice	*0.032	0.198	1,513 (0.806–2.843)
Abdominal pain	*0.000	*0.003	2.597 (1.387–4.865)	Vomiting	*0.000	*0.004	2,400 (1.319–4.368)
Viral load	*0.000	*0.005	1.406 (1.108–1.784)	Conjunctivitis	*0.004	*0.035	2.058 (1.052–4.024)

Logistic regression model was used for the univariate analysis. *P < 0.05

## Discussions

Jui government hospital in Freetown, Sierra Leone was approved as an EHC on Oct1, 2014 to receive suspected EVD patients. Once the EVD cases were confirmed, the patients were transferred to other ETCs such as P.T.S. 1, Kenema, 34 Military Hospital, Kerry Town and Goderich Hospital in Freetown. Altogether 152 confirmed patients were transferred to the above-mentioned ETCs. On Jan 1, 2015, Jui Government Hospital was approved as an ETC, and patients began to receive treatment in this institution thereafter. In the retrospective analysis, for the viral and clinical characteristics of the EVD patients, we used data from all patients with confirmed EVD at Jui Government Hospital from October 1, 2014 to March 21, 2015. The data included patients’ clinical presentations, time points when blood EBOV turned negative, durations of hospital stay, survival rates in different age groups, and other comparisons between survivors and non-survivors.

In the previous published reports, no detailed data was available on how soon the blood EBOV turned negative in EVD patients. Our observation revealed that blood EBOV turned negative very slowly—only 63.69 % patients turned blood EBOV negative in 3 weeks. This partially explained why EVD spread so widely and persisted for so long in West Africa. The surviving EVD patients in our study were discharged after the symptoms remained absent for 48 h, and the blood samples were negative for EBOV in two consecutive tests with quantitative RT-PCR assay. The mean hospitalization time for surviving patients was 20.38 ± 7.58 days. In other studies, the researchers continued to monitor EBOV RNA levels in sputum, saliva, conjunctiva swabs, stool, urine, and sweat using real-time RT-PCR assay after the blood EBOV RNA turned negative. They found that urine samples remained positive for EBOV RNA for as long as 13 days after blood turned negative,and sweat samples remained positive throughout the observation period for an additional 10 days (23 days after blood became EBOV RNA negative [7]). Another study reported that EBOV existed in cerebral spinal fluids and semen for much longer in EVD patients [8]. Even Varkey and colleagues described a patient who recovered from EVD and subsequently showed severe unilateral uveitis during convalescence. In this case, viable Zaire Ebola virus was detected in aqueous humour 14 weeks after the onset of EVD and 9 weeks after the clearance of EBOV from the blood [9]. Such findings warrant further investigations in the future. Although it was unclear whether or not such residual viruses were pathogenic, these findings suggested that when EVD patients are recovered and discharged, they should be put on continued quarantine for a period of time to avoid close contact with other people.

The Case Fatality Rates (CFR) of EVD was reported to be between 40 % and 74 % [10–17]. In our study, the total mortality rate was 48.77 % for confirmed cases, which was similar to that in the previous reports. As a trend observed in this outbreak, the fatality rate was more than 70 % during the early stage, and dropped to 40 %–60 % at a later stage. This may be attributable to the public’s poor awareness of this disease, the delay in seeking treatment, the inadequate measures for diagnosis and treatment, and the limited media coverage when the outbreak erupted. Later, as more awareness came from the international medical associations causing more international medical teams to arrive, the public became better educated and the patients started to receive more effective diagnosis and treatment. Thus at the later stage of the virus outbreak, early discovery, early diagnosis, and early treatment helped bring the fatality rate down.

We also made observations about the length of the hospital stay for the non-survivors. We found that most non-survivors died within 4 days after admission, which was consistent with other reports [10–17]. Only 10 % died after day 12 upon admission. This indicated that EVD has high degree of virulence. It also suggested that early diagnosis and early treatment is critical to the patients’ survival. Just as some scholars stated, the lack of public awareness of the disease as well as the lack of synergy among governments and international organizations contributed to the current epidemic [18]. The public should be educated and instructed to seek medical attention as early as they notice any symptoms, and the proactive treatment should be deployed as soon as the patients are admitted.

The age factor has been noted in the past EVD outbreaks and remains an important factor in the current outbreak [19–21]. The previous reports showed that older age was associated with worse outcomes, and the association between the two was often attributable to increased coexisting conditions in the elderly [10, 11, 14, 15]. In contrast, we did not find statistically significant differences between the age of non-survivors and survivors in our study (median age: 29 (IQR: 17–40) vs.27 (IQR: 17–35); average age: 29.32 ± 16.34 vs.28.97 ± 16.52). In order to determine the association between age and prognosis, we grouped the patients using different cutoffs, including median age (28), the cutoffs used in other studies (age 30, 40, 50), and multiple age groups at intervals of 5 years. In our comparison of the different age groups, no association was found between age group and prognosis. One distinction in our study was that the mortality rate for younger patients under the age of 6 was higher than the other age groups. This may be explained as follows: in the beginning of the EVD outbreak, adult patients encountered many complications such as diarrhoea-related electrolyte disorders and secondary infections, but soon received sufficient fluid therapy in many ETCs, so the mortality rate dropped among those patients. But for young children, since there was a shortage of paediatricians in many centres, the paediatric patients may not always have received sufficient attention or optimized therapy [22]. The young children were unable to care for themselves and their daily fluid intake was not always guaranteed while solely depending on the limited hospital resources. Additionally, poor access to IV vessels in paediatric patients may limit both the amount and the flow rate of fluid administered.

We also found that patients who presented the highest viral loads had the worst outcome, as had been the case for other strains of Ebola virus [10, 11, 16, 23]. Although the diagnostic value was excellent for postmortem swab samples [24], it was unclear how a Cycle Threshold (Ct) value from an oral swab correlates with that from a whole blood sample, therefore the viral load data for the oral swab samples (27/285) were not included in the analysis. The relationship between the viral load (copies of EBOV per milliliter) and the fatality rate was investigated. ROC curve analysis was plotted and a positive correlation was found between the two. A low viral load was associated with a better survival outcome, whereas a high viral load was an important indicator for fatality rate. Our finding was consistent with those of the previous reports [10, 11, 16, 23]. Based on this finding, it is plausible to assign patients with virus load of ≥10^6^ copies/ml to dedicated wards, where they can receive enhanced medical support and palliative care if resources allow, given their increased risk of death. It is noteworthy that the 27 cases for which EVD was confirmed using oral swab samples were all severely ill patients who died soon after admission. Exclusion of these cases may have introduced some bias in our results.

EVD was formerly named Ebola haemorrhagic fever, and bleeding is one of its hallmarks. Bleeding was noted among patients in previous outbreaks. However, in this outbreak, the reported bleeding rates ranged from 2.27 % [11] to 51.00 % [10]. In our study, only 35 of 285 patients (12.28 %) suffered from visible bleeding during their hospitalization, which is a relatively low number when comparing to other reports [25]. This suggested that bleeding may not be a major characteristic of EVD patients in this outbreak. Such discrepancy may be associated with the evolvement of the virus’s pathogenicity as its genes undergo mutations.

Finally, we analysed which factors were associated with patients’ outcomes. The incidences of various symptoms in EVD patients reported here are consistent with those of previous reports, with only minor differences. The most common EVD manifestations on admission were fever, weakness, loss of appetite, vomiting, cough, abdominal pain, headache, joint pain, and diarrhoea. In addition to factors such as age and viral load, previous studies described many other important factors associated with fatal outcomes, including hiccups, haemorrhagic signs, fever, weakness, dizziness, diarrhoea, myalgia, difficulty breathing, extreme fatigue, vomiting, mental symptoms, loss of appetite, confusion, and conjunctivitis [11–14, 16, 26]. The multivariate analyses in each study may include one or more of the aforementioned presentations. This indicated that: first, none of the clinical manifestations observed among patients in this outbreak was unique; and second, the way the patient history information was collected may vary in each study.

In our study, the multivariate analyses showed that EBOV viral load, abdominal pain, confusion, conjunctivitis, and vomiting were independently associated with the death outcome of EVD patients. It is understandable that high viral load is related to fatality rate, and that digestive discomfort may be associated with patients’ prognosis. In our study, the most powerful predictor of mortality in the multivariable regression model was confusion at admission (*P* value = 0.010), with an Odds Ratio of 4.150 (95 % *CI*: 1.401–12.293) favouring mortality. This finding indicated that the more severe the condition was at the time of admission, the higher the risk of death. We observed that due the lack of peculiar presentations of EVD and the less-developed health care capacity in Africa, many patients failed to seek medical attention promptly after they experienced some non-specific symptoms such as fever, thus delaying the diagnosis and treatment. Some patients died on their way to the hospital, some died upon arrival before the physicians had the opportunity to examine them, and yet some died on day 1 (11/285) or day 2 (23/285) after admission. In these circumstances, it was too late for the patients to receive proper treatment for such a severe disease. Therefore, we should raise the public’s awareness that early discovery, early diagnosis, and early treatment are essential.

Early proactive intervention can significantly improve the prognosis of the patients. In our clinical observations, we found that the clinical presentations in this EVD outbreak was primarily severe gastro enteric symptoms, such as nausea and lack of appetite accompanied by excessive vomiting and severe diarrhoea. Although due to the limitations of the medical equipment, we were unable to carry out laboratory tests to determine the cause of death for each non-survivor, we suspected that the loss of body fluids, abnormal metabolism, electrolytes imbalance, and hypovolemic shock, which were all secondary to the severe digestive tract disorder, may have been the immediate causes in the death of most non-surviving patients. The patients are usually extremely weak after the onset of the disease and cannot eat or drink by themselves; therefore it is essential for the medical staff, while taking precautions to protect themselves, to administer adequate fluids to the patients as early and as quickly as possible. Early initiation of proactive fluid therapy, particularly in the first days of hospitalization and even before the EBOV test results are received, is vital to saving patients’ lives and reducing fatality rate.

A few limitations exist in our study. First was the limitation of the data. There were many challenges regarding the collection of accurate clinical and epidemiological data on site in West Africa. Heavy workload, language barriers, and lack of an information technology infrastructure which would otherwise allow healthcare professionals to record data electronically at the point of patient contact, had all contributed to the incomplete data presented in our study.

The second limitation was that no analysis was available for the time period between symptom onset and admission, and in most cases it was difficult to pinpoint the exact date when symptom started. The majority of the patients were not able to recall or describe when the symptoms started; it was especially so for the more severe patients.

Another drawback is the limitations on the clinical observations. As we described previously, some patients were transferred to other ETCs before Jui Government Hospital became a designated ETC, and the treatment protocols in the other ETCs may not always be consistent with the EVD SOP.

GI losses and electrolyte disturbances may be the major causes of death in many EVD patients. Laboratory tests that provide comprehensive data on patients’ haematological and biochemical indicators can help delineate the cause of death. More importantly, it may also provide instructions for treatment, thus reducing fatality rate. Unfortunately, we did not have a cross-contamination-proof testing facility in place while our centre was serving as an EHC or in its early stage of serving as an ETC. Therefore, we were unable to determine whether the 139 non-survivors died of EVD complications, related co-infections, or some other causes. We only evaluated the severity of the illness by the clinical manifestations, and transfused more fluid for the patients with profound GI losses and those unable to take oral fluids or medications. The biochemical testing and the testing for co-infection with malaria were not launched until a later stage due to the limitation of laboratory facilities. Even then, such data was only collected from a limited number of patients. While the data was of some help to the treatment, it was not sufficient for a meta-analysis.

In addition, we were not able to analyse the association between the treatment protocols and the outcome. During the 6 months of treatment, three different health care teams rotated and the physicians were on 6–8 h shifts. The medical staff professionals had different ways of practicing, thus the medical history, symptoms and signs were recorded in different manners.

## Conclusions

In conclusion, for the patients confirmed with EVD, the survival rate was 51.23 %. Some surviving patients did not become blood EBOV negative until 4 weeks after admission or later. Most non-surviving patients died within 1 week after admission. Patients under the age of 6 years and those with high viral load had a higher fatality rate. Patients who presented confusion, vomiting, abdominal pain, and conjunctivitis at the time of admission were at higher risk of death. Such patients should be the priority of medical attention and should be put under intensive treatment, particularly during the first week of hospitalization.

## Additional file

Additional file 1: Multilingual abstracts in the six official working languages of the United Nations. (PDF 699 kb)

## Abbreviations

AUOC: Areas Under Operator Curve
BSL-3: Bio-safety Level 3
CDC: Center for Disease Control
CFR: Case Fatality Rates
CMT: Chinese Medical Team
EBOV: Blood Ebola Virus
EHC: Ebola Holding Center
ETC: Ebola Treatment Center
EVD: Ebola Virus Disease
GI: Gastrointestinal
IQR: Inter Quartile Range
NERC: National Ebola Response Center
PCR: Polymerase Chain Reaction
RNA: Ribose Nucleic Acid
ROC: Receiver Operating Characteristic
RT-PCR: Reverse Transcription-Polymerase Chain Reaction
SARS: Severe Acute Respiratory Syndrome
SOP: Standard Operating Procedures
WHO: World Health Organization
