# Supplementary material for: Clinical presentations and outcomes of patients with Ebola virus disease in Freetown, Sierra Leone
Source: Infect Dis Poverty. 2016 Nov 3;5:101. doi: 10.1186/s40249-016-0195-9 (PMC5094140; doi:10.1186/s40249-016-0195-9)
Supplement: Additional file 1: — Multilingual abstracts in the six official working languages of the United Nations. (PDF 699 kb) [file 40249_2016_195_MOESM1_ESM.pdf]

Translation of the abstract into the five official working languages of the United Nations

العروض السريرية ونتائج المرضى الذين يعانون من مرض فيروس الإيبولا في فريتاون، سيراليون

يينج جي جي، شيويه تشانج دوان، شو دونج جاو، لى لى تشن لى دونج جي ون كانج لى لى فو وانج يو هوا منج شياو يانج، بن فانج لينج، شيويه أي سونج، مى لى كو، تاو جيانج، شي كو م. كوروما، جيمس بتنجالي وهوى جوان دوان

ملخص
خلفية: تم جمع وتحليل بيانات سريرية ومخبرية من مرضى يعانون من مرض فيروس إيبولا في مستشفى جوي الحكومي في فريتاون، سيراليون، حيث تم استقبال المرضى الذين يعانون من مرض فيروس الإيبولا و / أو تم علاجهم من 1 أكتوبر 2014 إلى 21 مارس 2015 خلال تفشي مرض فيروس الإيبولا في غرب أفريقيا.
الطرق: شملت الدراسة 285 مريض تأكدت إصابتهم بمرض فيروس الإيبولا وتتبعتهم حتى نقطة النهاية (الشفاء أو الموت). وتأكدت الإصابة بمرض فيروس الإيبولا من خلال المقايسات الكمية RT-PCR التي تؤكد وجود فيروس الإيبولا في الدم.
النتائج: من بين الحالات الـ285 المؤكدة مخبريا للإصابة بمرض فيروس الإيبولا في مستشفى جوي الحكومة، 146 تعافى وتوفي 139، مع معدل البقاء على قيد الحياة 51.23٪. وكان معدل البقاء على قيد الحياة في المرضى الذين تقل أعمارهم عن 6 سنوات أقل (37.50٪). معظم غير الناجين (79.86٪) لقوا حتفهم خلال 7 أيام بعد دخول المستشفى وكان وقت المكوث في المستشفى لغير الناجين، 5.56 ± 6.11 يوما. في أكثر من نصف الناجين (63.69٪) تحول فيروس الإيبولا في الدم إلى سلبي في غضون 3 أسابيع بعد دخول المستشفى وكانت فترة المكوث في المستشفى للناجين 20.38 ± 7.58 يوما. تم العثور على حمل فيروسي مرتفع في الدم (≥106 نسخة / مل) ليكون منبئ بالوفاة كما يتضح من تحليل منحنى خاصية التشغيل المتلقي. وكان احتمال بقاء المرضى على قيد الحياة أقل من 15٪ عندما كان الحمل الفيروسي في الدم أكثر من 106 نسخة / مل. أظهرت التحليلات متعددة المتغيرات أن الحمل الفيروسي في الدم (P = 0.005)، الارتباك (P = 0.010)، ألم في البطن (P = 0.003)، التهاب الملتحمة (P = 0.035)، التقيؤ (P= 0.004) كانت عوامل المرتبطة بشكل مستقل مع نتائج المرضى المصابين بمرض فيروس الإيبولا.
الاستنتاجات: حدث معظم الوفيات في غضون أسبوع واحد بعد دخول المستشفى للعلاج، وكانت فرص المرضى في سن 6 سنوات أو أصغر أقل في البقاء على قيد الحياة. تحولت فيروس الإيبولا في الدم في معظم المرضى الذين بقوا على قيد الحياة إلى سلبي في غضون 1-4 أسابيع بعد دخول المستشفى. ارتبطت عوامل مثل ارتفاع الحمل الفيروسي في الدم، والارتباك، وألم في البطن والقيء والتهاب الملتحمة مع بسوء التشخيص للمرضى المصابين بمرض فيروس الإيبولا.

Translated from English version into Arabic by Mahmoud Sami, through

TRANSLATORS WITHOUT BORDERS

285 例埃博拉病毒病患者的临床特征及预后分析

吉英杰， 段学章，高旭东，李雷，李晨，纪冬，李文刚，王立福，孟玉华，杨晓，凌宾芳，宋雪艾，顾梅蕾，江涛，Sheku M. Koroma, James Bangalie, 段惠娟

摘要
引言：西非埃博拉爆发期间，2014 年 10 月 1 日至 2015 年 3 月 21 日，塞拉利昂弗里敦中塞友好医院收治了大量的埃博拉病毒病（EVD）患者，我们收集和分析了这些患者的临床和实验室数据，分析了其临床特征和预后。
方法：所有确诊的 EVD 患者均通过 RT-PCR 的检测得以确认，共有 285 例 EVD 确诊患者纳入本研究。
结果：285 例确诊 EVD 患者中，146 例康复，139 例死亡，整体存活率 51.23%，其中 6 岁以下患者存活率较低（37.50%）。死亡患者中大部分（79.86%）患者在入院 7 天内死亡，其平均住院时间为 5.56±6.11 天。一半以上（63.69%）的康复者的血清 EBOV 在入院后 3 周内

转阴，其平均住院时间为 20.38±7.58 天。ROC 曲线分析结果显示，高病毒载量预示着患者的死亡，血液病毒载量$\geq 10^6$copies/ml 的患者存活的几率低于 15%。与生存率相关的多因素分析显示：高病毒载量 ($P = 0.005$)、意识障碍($P = 0.010$)、腹痛($P = 0.003$)、结膜炎(P =0.035) 和呕吐($P = 0.004$)是与患者死亡密切相关的风险因素。
结论：EVD 患者的死亡多发生在入院后 7 天内，6 岁以下患者病死率更高。EVD 存活患者的血液病毒载量大多在入院后 1-4 周内转阴。高病毒量、意识障碍、腹痛、结膜炎和呕吐等表现预示患者的预后差。

Translated from English version into Chinese by Ying-Jie Ji

Tableau clinique et évolution des patients porteurs de la fièvre d'Ébola à Freetown (Sierra Leone)

Ying-Jie Ji, Xue-Zhang Duan, Xu-Dong Gao, Lei Li, Chen Li, Dong Ji, Wen-Gang Li, Li-Fu Wang, Yu-Hua Meng, Xiao Yang, Bin-Fang Ling, Xue-Ai Song, Mei-Lei Gu, Tao Jiang, She-Ku M. Koroma, James Bangalie et Hui-Juan Duan

Résumé
Contexte: Des données cliniques et de laboratoire ont été recueillies sur des patients porteurs de la fièvre d'Ébola et analysées au Jui Government Hospital de Freetown, au Sierra Leone, où ces patients ont été reçus et/ou traités entre le 1er octobre et le 21 mars 2015, au cours de l'épidémie d'Ébola en Afrique de l'Ouest.
Méthodes: L'étude a inclus 285 patients présentant une fièvre d'Ébola confirmée, qui ont été suivi jusqu'au terme de leur évolution (guérison ou décès). La contamination par le virus d'Ébola (VEBO) été confirmée par la détection du virus dans le sang par RT-PCR.
Résultats: Sur les 285 cas de fièvre d'Ébola confirmés par RT-PCR à l'hôpital Jui, 146 ont guéri et 139 sont décédés, soit un taux global de survie de 51,23 %. Le taux de survie était plus faible parmi les patients âgés de moins de 6 ans (37,50 %). La plupart (79,86 %) des patients qui n'ont pas survécu sont décédés dans les 7 jours suivant leur admission, avec une durée moyenne d'hospitalisation de $5{,}56 \pm 6{,}11$ jours. Plus de la moitié (63,69 %) des survivants avaient une sérologie VEBO négative 3 semaines au maximum après leur admission et leur durée moyenne d'hospitalisation était de $20{,}38 \pm 7{,}58$ jours. Une charge virale élevée ($\geq 10^6$ copies/ml) s'est avérée prédictive du décès, comme l'indique l'analyse des courbes de fonction d'efficacité du récepteur (ROC). La probabilité de survie des patients était inférieure à 15 % si la charge virale dépassait 10^6 copies/ml de sang. Les analyses multivariées ont montré que la charge virale sanguine ($P = 0{,}005$), un état de confusion ($P = 0{,}010$), des douleurs abdominales ($P = 0{,}003$), une conjonctivite ($P = 0{,}035$), et des vomissements ($P = 0{,}004$) étaient autant de facteurs associés, indépendamment les uns des autres, à l'issue de la maladie.
Conclusions: La plupart des décès sont survenus dans la semaine suivant l'admission. Le taux de survie était plus faible parmi les patients âgés de moins de 6 ans. La plupart des patients qui ont survécu sont devenus séronégatifs pour le VEBO entre 1 et 4 semaines après leur hospitalisation. Une charge virale élevée, un état de confusion, des douleurs abdominales, des vomissements et une conjonctivite représentaient des facteurs de mauvais pronostic pour les patients atteints de la fièvre d'Ébola.

Translated from English version into French by Suzanne Assenat, through

TRANSLATORS
WITHOUT BORDERS

Клинические проявления и исходы больных вирусной инфекцией Эбола во Фритауне, Сьерра-Леоне

Инцзе Цзи (Ying-Jie Ji), Сюэчжан Дуань (Xue-Zhang Duan), Сюйдун Гао (Xu-Dong Gao), Лэй Ли (Lei Li), Чэнь Ли (Chen Li), Дун Цзи (Dong Ji), Вэньган Ли (Wen-Gang Li), Лифу Ван (Li-Fu Wang), Юйхуа Мэн (Yu-Hua Meng), Сяо Ян (Xiao Yang), Биньфан Лин (Bin-Fang Ling), Сюэай Сун (Xue-Ai Song), Мэйлэй Гу (Mei-Lei Gu), Тао Цзян (Tao Jiang), Шэку М. Корома (She-Ku M. Koroma), Джеймс Бангали (James Bangalie) и Хуэйцзюань Дуань (Hui-Juan Duan)

Реферат

Предыстория: Были собраны и проанализированы данные лабораторных и клинических исследований больных вирусной инфекцией Эбола (EVD), проходивших лечение в государственной больнице Жуи (Jui Government Hospital) во Фритауне, Сьерра-Леоне, с 1 октября 2014 г. по 21 марта 2015 г. в период вспышки вируса Эбола в Западной Африке.

Методы: Было обследовано 285 больных с подтвержденным диагнозом вирусной инфекции Эбола, с последующим врачебным наблюдением до конечной стадии болезни (выздоровление или смертельный исход). Вирусное заболевание Эбола подтверждено анализами количественной ПЦР, определившими наличие вируса Эбола в крови.

Результаты: Из 285 случаев вирусной инфекции Эбола в государственной больнице Жуи, подтвержденных лабораторными анализами, выздоровело 164 больных и умерло139; при этом коэффициент выживаемости составил 51,23%. Более низкий коэффициент выживаемости наблюдался у больных моложе шести лет. Большинство невыживших (79,86%) умерли в течение семи дней после приема в больницу, при этом средняя продолжительность госпитализации невыживших составила 5,56±6,11 дня. В течение трех недель после поступления в больницу у более половины выживших (63,69%) анализ крови на вирус Эбола дал отрицательный результат, при этом средняя продолжительность госпитализации выживших составила 20,38±7,58 дня. Анализ с применением РОС-кривых выявил, что высокая вирусная нагрузка крови (($\geq 10^6$ копий/мл) является показателем невыживаемости. При вирусной нагрузке крови более 10^6 копий/мл вероятность выживаемости больного составляет менее 15%. Многомерные анализы показали, что вирусная нагрузка крови (P = 0.005), спутанность сознания (P = 0.010), боль в животе (P = 0.003), конъюнктивит (P = 0.035) и рвота (P = 0.004) явились факторами, независимо друг от друга связанными с исходами больных вирусной инфекцией Эбола.

Выводы: Большинство смертельных исходов произошло в течение одной недели после поступления в больницу, причем у больных моложе шести лет наблюдался более низкий коэффициент выживаемости. Большинство выживших больных показали отрицательный результат на вирус Эбола в крови в течение 1-4 недель после поступления в больницу. Такие факторы как высокая вирусная нагрузка крови, спутанность сознания, боль в животе, рвота и конъюнктивит были сопряжены с неблагоприятным прогнозом для больных вирусной инфекцией Эбола.

Translated from English version into Russian by Tatyana Johnson, through

Presentación y resultados en Pacientes con la enfermedad del virus Ébola en Freetown, Sierra Leona

Ying-Jie Ji, Xue-Zhang Duan, Xu-Dong Gao, Lei Li, Chen Li, Dong Ji, Wen-Gang Li, Li-Fu Wang, Yu-Hua Meng, Xiao Yang, Bin-Fang Ling, Xue-Ai Song, Mei-Lei Gu, Tao Jiang, She-Ku M. Koroma, James Bangalie and Hui-Juan Duan

Resumen

Antecedentes: Se recolectaron y analizaron datos clínicos y de laboratorio de pacientes con la enfermedad del virus del Ébola (EVD) en el Hospital Público Jui, en Freetown, Sierra Leona, donde, pacientes con EVD fueron recibidos y/o tratados desde el 1 de octubre de 2014 al 21 de marzo de 2015, durante el brote de EVD en África Occidental.

Métodos: Fueron admitidos en el estudio 285 pacientes con la EVD confirmada y se les realizó un seguimiento hasta el *punto final* (recuperación o fallecimiento). La EVD fue confirmada por pruebas de RT-PCR cuantitativas para detectar el virus de Ébola en la sangre (EBOV).

Resultados: Entre los 285 casos de la EVD confirmados en el laboratorio en el Hospital Público de Jui, 146 se recuperaron y 139 murieron, con una tasa de supervivencia global del 51,23%. Los pacientes menores de 6 años tuvieron una tasa de supervivencia más baja (37,50%). La mayoría de los no sobrevivientes (79,86%) murieron dentro de los 7 días siguientes a la admisión y el tiempo medio de hospitalización para los no supervivientes fue de 5,56 ± 6,11 días. Más de la mitad de los supervivientes (63,69%) resultaron con EBOV negativo en sangre dentro de las 3 semanas siguientes a la admisión y el tiempo medio de hospitalización para los supervivientes fue 20,38 ± 7,58 días. Se encontró predictivo de los resultados de no supervivencia, una alta carga viral en la sangre (≥106 copias/ml), como se indica por el análisis de la curva Reciever Operating Characteristic (ROC) (Característica Operativa del Receptor). La probabilidad de supervivencia de los pacientes fue de menos del 15% cuando la carga viral en sangre fue mayor de 106 copias/ml. Los análisis multivariables mostraron que la carga viral en sangre (P = 0,005), la confusión (P = 0,010), el dolor abdominal (P = 0,003), la conjuntivitis (P = 0,035) y los vómitos (P = 0,004) fueron factores asociados, de forma independiente, con los resultados de los pacientes con la EVD.

Conclusiones: La mayoría de los fallecimientos ocurrieron dentro de la semana siguiente a la admisión y los pacientes de 6 años o menores tuvieron una tasa de supervivencia más baja. La mayoría de los pacientes que sobrevivieron mostraron EBOV en sangre negativo dentro de 1-4 semanas después de la admisión. Factores como una alta carga viral en la sangre, confusión, dolor abdominal, vómitos y conjuntivitis se asocian a un peor pronóstico para los pacientes con la EVD.

Translated from English version into Spanish by patriciacassoni, through
